# Virtual Reality Exposure Therapy for Treating Fear of Contamination Disorders: A Systematic Review of Healthy and Clinical Populations

**DOI:** 10.3390/brainsci14050510

**Published:** 2024-05-17

**Authors:** Francesca Ferraioli, Laura Culicetto, Luca Cecchetti, Alessandra Falzone, Francesco Tomaiuolo, Angelo Quartarone, Carmelo Mario Vicario

**Affiliations:** 1 Department of Cognitive Science, University of Messina, 98121 Messina, Italy; laura.culicetto@studenti.unime.it (L.C.); alessandra.falzone@unime.it (A.F.); 2 IMT School for Advanced Studies Lucca, 55100 Lucca, Italy; luca.cecchetti@imtlucca.it; 3 Department of Clinical and Experimental Medicine, University of Messina, 98122 Messina, Italy; ftomaiuolo@unime.it; 4 Istituto di Ricovero e Cura a Carattere Scientifico (IRCCS) Centro Neurolesi Bonino-Pulejo, 98124 Messina, Italy; angelo.quartarone@irccsme.it

**Keywords:** immersive virtual reality exposure therapy, disgust, fear of contamination, OCD, specific phobia

## Abstract

Virtual Reality Exposure Therapy (VRET), particularly immersive Virtual Reality Exposure Therapy (iVRET), has gained attraction as an innovative approach in exposure therapy (ET), notably for some anxiety disorders with a fear of contamination component, such as spider phobia (SP) and obsessive–compulsive disorder (OCD). This systematic work investigates iVRET’s effectiveness in modulating disgust emotion—a shared aberrant feature across these disorders. Recent reviews have evaluated VRET’s efficacy against in vivo ET. However, emerging evidence also highlights iVRET’s potential in diminishing atypical disgust and related avoidance behaviors, expanding beyond traditional fear-focused outcomes. Our systematic synthesis, adhering to PRISMA guidelines, aims to fill this gap by assessing iVRET’s efficacy in regulating disgust emotion within both clinical and at-risk populations, identified through standardized questionnaires and subjective disgust ratings. This research analyzes data from eight studies on clinical populations and five on healthy populations, offering an insight into iVRET’s potential to mitigate the aberrant disgust response, a common transdiagnostic feature in varied psychopathologies. The findings support iVRET’s clinical relevance in disgust management, providing evidence for a broader therapeutic application of iVRET and pointing out the need for more focused and complete investigations in this emergent field.

## 1. Introduction

Exposure-based interventions are commonly used in the treatment of Specific Phobia (SP) but also other anxiety disorders [1,2], with special regard to fear-of-contamination-based disorders such as obsessive–compulsive disorder (OCD).

In DSM-5 [3], OCD was excluded from anxiety disorders and included in a separate category; further, recent clinical studies stress the importance of adding evaluation of fear of contamination for a better metacognitive evaluation of OCD [4,5]. Interestingly, a study by Ojserkis et al. [5] explores the concept of mental contamination and the role of disgust in the context of OCD and post-traumatic stress disorder (PTSD), highlighting how mental contamination and disgust are implicated in these conditions. The study found that mental contamination can partially mediate the relationship between disgust propensity (the ease with which an individual feels disgusted) and the severity of contamination-based OCD symptoms. This suggests that a higher propensity to experience disgust may contribute to the maintenance of OCD symptoms by frequently eliciting feelings of disgust in response to both external and internal stimuli, leading to mental contamination and subsequent washing behavior.

Notably, SP and OCD share an aberrant sense of disgust emotion, as we observed in a very recent narrative synthesis on disgust as a transdiagnostic index in a large population of clinical and neurologic disorders [6].

In recent years, the application of virtual reality technology in the clinical field is growing, thanks to its relevance as a tool for constructing experimental environments pertinent to the investigation of clinical symptoms. An example of this application is evident in Pavlovian conditioning protocols [7,8], where VR is utilized to establish scenarios conducive to the examination of fear learning and extinction. These mechanisms are pivotal in understanding anxiety and associated disorders, including PTSD.

Further clinical application of VR includes psychotherapy such as exposure therapy (ET) [9,10,11]. It complements traditional exposure-based interventions, offering real-time interaction in a computer-generated 3D environment, making it a valuable tool in addressing specific phobias [12,13] and other psychopathologies [14,15,16].

The classic exposure therapy approach, as well as recent approaches that implement VRET, have a strong impact on the extinction of this aberrant sense of disgust, as reported by several studies on clinical populations [17,18,19,20,21,22,23,24] and even on at-risk healthy populations [25,26,27,28,29]. VRET uses virtual reality technology to expose patients to situations that may trigger feelings of disgust, helping them manage their emotions. It is thought that by exposing individuals to these controlled simulations, they can develop coping mechanisms and reduce avoidance behavior [30].

Research on VRET and disgust is still limited, but it is an emerging field. Few studies focus on the efficacy of VRET on regulating disgust emotion, while the majority investigate the efficacy of this treatment on general anxiety symptoms or principally on the fear emotion. Further, most recent systematic reviews and meta-analyses are limited to evaluating the efficacy of VRET in comparison to in vivo ET [1,13,14,31,32,33].

Therefore, taking into account the numerous pieces of evidence on the advantages of using VRET over in vivo ET, in particular immersive VRET (iVRET), along with evidence of the linkage to abnormal disgust emotion and several mental disorders, our aim is to conduct a systematic literature synthesis to evaluate the effectiveness of treating an aberrant sense of disgust through iVRET in clinical populations where this technique is most used, i.e., SP and OCD, as well as on at-risk populations.

This has important clinical value as it would constitute additional evidence in favor of the treatment of disgust as a key symptom of several clinical conditions and in addition it returns a comprehensive evaluation of a promising new therapeutic approach that exploits immersive virtual reality.

## 2. Materials and Methods

The study’s search, identification, and selection process was performed according to PRISMA guidelines [34]. 

### 2.1. Study Search and Selection

**Figure 1:**
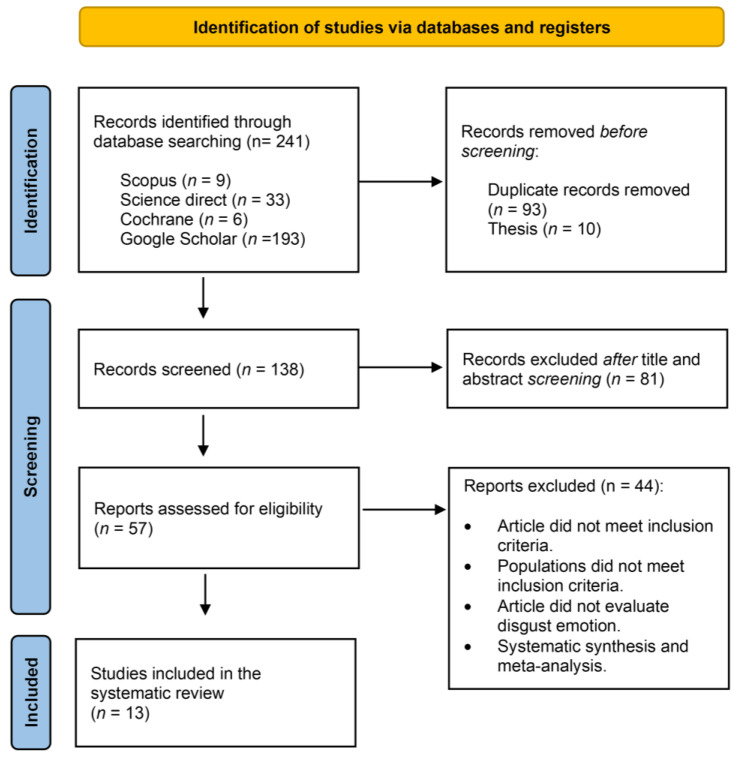
PRISMA diagram flow.

A comprehensive search was conducted from September to November 2023 across multiple databases, including Google Scholar, PubMed, ScienceDirect, Cochrane, and Scopus, using a combination of keywords, including “Virtual Reality Exposure Therapy”, “Immersive Virtual Reality Exposure Therapy”, “VRET”, “Disgust”, “Disgust Emotion”, “Fear-of-Contamination Disorders”, “OCD”, and “Specific Phobia”. The identification process involved the removal of duplicates, topic screening based only on the title and abstract, and a thorough assessment for eligibility criteria. All of the study search and identification processes were conducted independently by two researchers (FF and LC) in order to reduce the risk of bias (i.e., publication bias, time lag bias, and language bias); any disagreement was further discussed by the two authors. As shown in Figure 1, a total of 241 records were identified, with 138 records screened, and 57 assessed for eligibility. The final selection included 13 studies, 8 on clinical populations and 5 on heathy populations.

### 2.2. Eligibility Criteria 

We included studies based on the following eligibility criteria: (a) studies published in the last 10 years to capture the most recent VRET interventions in (b) RCTs and non-RCTs, in which (c) the clinical population meets the diagnostic criteria for SP and OCD, (d) or in which healthy populations were grouped by disgust or phobia ratings, and (e) received an iVRET intervention that (f) was compared with a control group or condition, as well as (g) single case studies on a population of interest (i.e., meeting c and d criteria). Further, for the purpose of our analysis, we focus on trials that (h) evaluate disgust emotion through standardized questionnaires, (i) or subjective ratings of disgust, as well as (j) the reduction of avoidance behavior.

Moreover, according to the PICO framework [35], items collection focused on populations of interest, intervention type, study characteristics, and outcome measures to evaluate the efficacy of iVRET in regulating disgust emotion (for a detailed description see Table 1).

Further, where possible and if not already reported in the publication, the calculation of effect sizes (Cohen’s *d* [36]) was employed to quantify the impact of iVRET interventions on disgust responses. Following suggestions by Harrer et al. [37], since correlation data between pre- and post-interventions were not available or computable for all collected studies, we quantified the effect of iVRET interventions on disgust emotion by comparing experimental and control groups in post-intervention sessions, or we derived it from other reported effect size measures (i.e., *η*^2^).

**Table 1:** Definitions of used criteria for the items’ inclusions according to PICO guidelines (Mattos and Ruellas, 2015) [35].

PICO	Definition
Population	A clinical population that meets the diagnostic criteria for SP and OCD, according to DSM-5/5-TR [3,38] or the Composite International Diagnostic Interview (CIDI [39]). A healthy population sampled based on a high/low fear of contamination or a specific phobia, in items where there is a presence of OCD symptoms (Yale–Brown Obsessive–Compulsive Scale II, YBOCS-II [40]; Obsessive–Compulsive Inventory Revised, OCI-R [41]; or SP (i.e., Fear of Spiders Questionnaire, FSQ [42]) were assessed).
Intervention Type	iVRET designed for treating fear of contamination and specific phobias.
Study Characteristics	We collected RCTs and non-RCTs as well as single case studies where it was possible evaluate the effectiveness of iVRET on disgust emotion.
Outcomes	Disgust sensitivity was measured by standardized questionnaires (i.e., Fragebogen zur Erfassung der Ekelempfindlichkeit, FEE [43]; Disgust Scale-Revised, DS-R [44]; Food Disgust Scale, FDS short [45] or Likert’s scales (10 or 100 point); or Subjective Units of Distress (SUDs [46]); as well as Self-Assessment Manikin (SAM [47])). Trait Disgust was measured by standardized questionnaires (i.e., Ekel-State-Fragebogen, ESF [48]). Behavioral Avoidance was measured by a behavioral approach test (BAT [29]).

The effect sizes (*d*) were calculated for between-group design studies according to the following formula [37]:

$$d=\frac{Mg1-Mg2}{Pooled\;SE}$$

$$Pooled\;SE=Spooled\;\sqrt{\frac{1}{n_{1}}+\frac{1}{n_{2}}}$$

$$Spooled=\sqrt{\frac{{(n}_{1}-1)s_{1}^{2}+{(n}_{2}-1)s_{2}^{2}}{{(n}_{1}-1)+{(n}_{2}-1)}}$$

We derived Cohen’s *d* from *η*^2^ according to the following formula [36]:

$$f=\sqrt{\frac{\eta^{2}}{1-\eta^{2}}}$$

d=2f

Cohen’s *d* and metrics of interest for all collected studies are reported in Table 2.

## 3. Results

### 3.1. Results in Heathy Populations

The collected evidence from selected studies on heathy populations (see Table 2 below, top panel) investigates the effectiveness of iVRET in addressing disgust, particularly in the context of contamination fear [25,26,27,28,29].

The two studies by Inozu and coworkers [25,26] explore the efficacy of iVRET in reducing contamination fear, disgust, and the urge to wash hands. The first study focuses on individuals with a high contamination fear [25], while the second study examines individuals with varying levels of contamination fear [26]. In both studies, VR scenarios were designed based on research conducted by Belloch and colleagues [20]. They took place in a kitchen setting where the degree of dirtiness and disgust gradually increased from Scenario 1 to Scenario 4; further, participants rated their levels of anxiety, disgust, and urge to wash hands before and after each VR session. The studies specifically highlighted the role of iVRET in reducing feelings of disgust. Specifically, authors [25] show significant decreases in post-test scores for anxiety, disgust, and the urge to wash hands in the experimental group compared to their pre-test scores. The interaction effect between time and group was significant for all contamination-related ratings, including disgust, indicating that iVRET not only reduces anxiety and behavioral anomalies but also disgust sensitivity scores. These findings were replicated in a second study [26] where authors found significant effects of dirtiness and group (high and low contamination fear) on anxiety, disgust, and the urge to wash; interestingly, mediation analysis showed that disgust mediated the relationship between contamination fear and the urge to wash. This finding highlights the potential role of disgust in the etiology and phenomenology of contamination and washing symptoms. Furthermore, in both studies by Inozu [25,26], we found a very large effect size for the efficacy of the iVRET used on disgust outcomes, as reported in Table 2. 

Ammann et al. [27] investigated whether VR could successfully evoke disgust and if this emotion could influence participants’ willingness to eat chocolate in a VR environment. In particular, participants were exposed to a VR environment where they either saw a neutral scenario or a disgust-inducing scenario (a dog defecating what appeared to be chocolate); then, they were asked to eat real chocolate in the real world. The authors found that participants exposed to a disgust-inducing scenario were less likely to eat the chocolate compared to those in the control condition. This study assessed changes in disgust sensitivity and its influence on behavior, enhancing the understanding of how disgust sensitivity influences behavior in a virtual context. Additionally, a mediation analysis revealed that the relationship between food disgust sensitivity and willingness to eat chocolate was mediated by the participants’ sense of physical presence in the VR environment.

These results underscore VR’s potential as a tool for studying and potentially modifying disgust-related behaviors, especially in contexts where physical presence plays a significant role. 

The study from Dozio et al. [28] explores the design of virtual environments to elicit specific emotions, including disgust. The results highlighted significant emotional responses, including disgust, measured using self-assessment methods like the SAM questionnaire. The study evaluated the effectiveness of VR environments in eliciting disgust and other emotions, but it does not compare disgust levels pre- and post-intervention. However, even if it does not directly address therapeutic applications, its findings could inform the design of VR-based therapy targeting specific emotional responses.

Finally, the study by Minns et al. [29] demonstrated that iVRET significantly reduced spider fear, as evidenced by improvements in both FSQ and behavioral approach test (BAT) scores. A large effect size (Cohen’s *d* = 0.85) for the FSQ and a medium effect size (Cohen’s *d* = 0.47) for the BAT were observed, indicating substantial improvements in spider fear from pre- to post-treatment. Regarding disgust emotion, participants showed that the highest level of disgust was felt during VRET, but detailed statistical results focusing solely on disgust were not explicitly discussed. Thus, this study offers preliminary evidence supporting the effectiveness of iVRET in phobia treatment. It demonstrates significant reductions in fear and avoidance behaviors following treatment. However, future research is needed to investigate its specific impact on disgust emotion and to validate these findings in clinical populations.

### 3.2. Results in Clinical Populations

Collected studies on clinical populations are listed in Table 2, bottom panel, sub-divided into SP and OCD items.

### 3.3. Specific Phobias (SP)

Anxiety disorders are the most prevalent mental disorders, impacting 14% of the population [49]. Unlike typical feelings of excitement or nervousness, pathological fear is characterized by terror and anxiety levels that are disproportionate to the actual danger. According to DSM-5-TR [39], SP is characterized by an excessive and persistent fear of a specific object, situation, or activity that is generally not harmful. SP, with a prevalence rate of 6.4%, is the most common type of anxiety disorder [38].

Recent advancements in VR technology have opened new avenues for innovative approaches to ET for SP [50]. Among these, spider phobia, or arachnophobia, has emerged as a key target for VR-based treatment due to its prevalence and the controlled environment VR provides. Our article provides a review of several studies that have utilized VR to varying degrees and with different methods aimed at understanding and reducing the fear response in individuals with spider phobia. From automated immersive experiences to the application of tactile and haptic feedback, the selected studies represent the forefront of technology-assisted therapy in managing SP. Binder et al. [18] highlights significant avoidance behavior in individuals with SP when exposed to virtual simulations of spiders. Through iVR tasks designed to measure avoidance, such as “Fishing” (a behavioral search task), “Path-Choice” (a forced-choice task), and “Touch the Enemy” (a behavioral approach task), the study observed that phobic participants exhibited pronounced avoidance behaviors and physiological responses indicative of negative emotions (i.e., fear and disgust). These responses included increased pupil size and heart rate when approaching or being near virtual spiders.

This study demonstrated effectiveness in reducing avoidance behaviors and physiological activation related to spider phobia. Although differences across scenarios in disgust emotion were not statistically analyzed, disgust sensitivity was measured through the FEE [41]. The study found a significant positive correlation between the FEE total score and FSQ [42]. Specifically, this positive correlation was also significant for the scale sub-category body secretion and hygiene. Interestingly, the FEE sub-category oral rejection negatively correlates with spider valence. Taken together, these findings suggest a significant reduction in disgust proportional to the reduction in fear of spiders following iVRET. Diemer et al. [24] explored the effects of quetiapine XR, an antipsychotic medication, in individuals with arachnophobia by administering a single dose before a VR challenge simulating a spider encounter. At baseline, participants completed assessments of disgust sensitivity (FEE [41]) and anxiety sensitivity (Anxiety Sensitivity Index, ASI [51]), and their electrodermal activity (EDA) was measured. While disgust sensitivity was not assessed post-intervention, the authors observed significant changes in behavioral avoidance and electrodermal responses according to the VR challenge. 

Further, the quetiapine group exhibited significant reductions in anxiety, particularly in somatic symptoms, along with a notable decrease in skin conductance, a physiological anxiety marker. These findings indicate that VRET was feasible in inducing the required emotional responses for treating OCD.

Tardif et al. [19] aimed to enhance our understanding of the psychological mechanisms underlying VR exposure therapy, particularly focusing on the role of tactile and haptic feedback. A sample of 59 participants were randomly assigned to experience either visual-only stimuli, visual plus tactile stimuli, or visual plus tactile and haptic feedback stimuli during VR exposure. The study found that changes in beliefs about spiders and self-efficacy were the only significant predictors of phobia reduction, questioning the additional clinical benefit of tactile and haptic feedback in VR settings. Trait disgust was measured using the ESF [44], but changes in disgust did not significantly predict the reduction in fear of spiders; however, a large effect size (Cohen’s *d* = 1.70) was reported for the efficacy of iVRET on ESF differences pre- and post-treatment, indicating a strong impact of iVRET in regulating disgust emotion. In summary, the collected studies suggest that iVRET emerges as a promising intervention for SP, effectively reducing both disgust and fear, which are crucial emotional components of conditions like arachnophobia. The therapy’s success in diminishing avoidance behaviors and physiological responses suggests its potential as a significant tool in phobia treatment. However, variability in its impact on disgust across different studies indicates the need for further exploration to enhance VRET’s efficacy and tailor it to individual patient needs. This underscores the importance of considering cognitive and self-efficacy factors in determining its therapeutic effectiveness.

### 3.4. Obsessive–Compulsive Disorder (OCD)

OCD is a debilitating condition with a global lifetime prevalence of approximately 2 or 3% (Abramowitz et al., 2009 [52]). It is characterized by obsessions, which are recurrent, persistent, and intrusive, and unwelcome thoughts and compulsions, which are repetitive behaviors or mental actions carried out in a stringent, ritualistic manner, or a combination of both types of symptoms [39]. 

The advent of VRET has provided a novel avenue for the treatment of OCD, offering patients a safe yet evocative environment in which to confront their fears. This synthesis of research explores the efficacy and patient acceptance of VRET across various studies, with particular attention to the evocation of disgust and anxiety within contamination-related OCD. 

The study by Belloch et al. [20] examines the use of iVRET and its application for patients with OCD, with a particular focus on its effectiveness from the patients’ perspective. In this study, four women diagnosed with OCD were exposed to various scenarios within a Contaminated Virtual Environment (COVE). The patients evaluated their sense of presence, emotional engagement, and reality judgment, as well as the levels of anxiety and disgust they experienced while performing tasks in the COVE. The results indicated that the COVE effectively generated a strong sense of presence for the participants. Furthermore, it was observed that as the level of virtual contamination increased, the participants’ level of anxiety and disgust also increased. Additionally, the study found that the anxiety experienced by the participants correlated with levels of emotional engagement and sense of presence within the virtual environment. Based on these findings, the authors concluded that the virtual environments used in the study were effective in eliciting the necessary emotional responses for OCD treatment.

In a single case study [21], the practicality and efficacy of personalized iVRET was investigated, where 360° videos were examined for the treatment of a contamination/washing OCD patient. A patient with severe and treatment-resistant OCD underwent 15 weekly VR sessions wearing a VR headset, during which she was exposed to immersive videos captured from her own point of view in familiar settings. The treatment was well-received, with the patient experiencing significant therapeutic benefits. These included reduced emotional reactivity as indicated by skin conductance levels, a decrease in OCD symptoms, and enhanced quality of life.

Initially, the patient exhibited adverse reactions to the anxiety-inducing content presented in the videos, such as feelings of disgust and a tendency to avoid looking at distressing elements. However, as the therapy progressed, these avoidance behaviors diminished, indicating successful adaptation. Clinically, the patient demonstrated improvement, as evidenced by a five-point reduction on the Y-BOCS score [40], alleviation of depression symptoms, and an expanded range of activities in daily life. This progress suggests that the therapeutic effects extended beyond the specific scenarios depicted in the virtual reality session. Although this study did not specify statistical results directly related to disgust, the overall reduction in OCD symptoms and enhancement in the patient’s ability to engage with previously avoided scenarios indirectly imply a decrease in disgust responses.

In another study [22], the aim was to validate VR’s effectiveness compared to the current first-line treatment of in vivo exposure and response prevention (ERP), particularly for patients with contamination-related OCD. The study sought to assess the comparability of VR and in vivo ERP sessions across various measures, including self-reported anxiety, therapeutic alliance, exposure engagement, and psychophysiological indicators of emotional response. The results showed significant increases in SUDs (Wolpe and Lazarus, 1966) across an exposure hierarchy, demonstrating that both VR and in vivo sessions elicited similar anxiety profiles. Regarding disgust emotion, this increase in SUDs across exposure tasks suggests that iVRET may also impact disgust, given its relationship with anxiety in OCD, although this was not directly measured. Moreover, there were no significant differences between the two methods in terms of pre- and post-session anxiety levels. Furthermore, the study found that VR was beneficial for participant engagement and adherence to exposure tasks.

The primary objective of the study conducted by Fajnerová et al. [23] was to assess the effectiveness of a set of standardized stimuli designed to represent OCD subtypes (i.e., contamination/cleaning, checking, symmetry, and hoarding) within a virtual environment reproducing a home in provoking anxiety and symptoms of OCD in patients compared to a to a group of healthy controls. Trait disgust was assessed through distress level measured by SUD scores, which was only measured post-intervention. Statistical analysis revealed significant differences in distress and compulsive behaviors between OCD patients and healthy controls, suggesting the potential of VR for symptom provocation.

Miegel et al. [17] aimed to investigate the use of iVRET with a protocol implementing response prevention (VERP) in treating patients with contamination-related OCD (C-OCD), primarily experiencing disgust. Patients underwent VERP treatment over six weeks, involving four consecutive exposure sessions. The study assessed various parameters, including subjective distress (by SUD score), physiological arousal (heart rate and skin conductivity), sense of presence in virtual reality, and simulator sickness.

Subjective distress, serving as a proxy for disgust, was successfully induced in six out of eight patients during the first exposure session, with significant increases in SUD ratings in session 3 compared to baseline. This suggests that VERP can effectively provoke disgust in patients with C-OCD, thereby contributing to the therapeutic process by exposing patients to feared stimuli in a controlled manner. However, the persistence of obsessions, a moderate sense of immersion in the virtual environment, and varied individual responses suggest significant room for improvement. The reasons behind these mixed outcomes, including the possibility of disgust being more treatment-resistant, potential sample selection biases, or aspects of the VR environment itself remain areas for further investigation.

The body of research reviewed herein underscores the potential of iVRET as a promising avenue for treating OCD, particularly addressing contamination-related symptoms with significant impacts on disgust and anxiety. Studies reveal that VRET can evoke strong emotional responses, such as anxiety and disgust, provoking a realistic yet safe environment for patients to confront their fears. The effectiveness of VRET in reducing OCD symptoms, including emotional reactivity and compulsive behaviors, highlights its potential as a complementary or alternative therapy to traditional methods. This emerging evidence supports VRET’s role in enhancing patient engagement, reducing avoidance behaviors, and improving quality of life for those with OCD, suggesting a valuable addition to existing treatment modalities.

## 4. Discussion

This systematic review highlights iVRET’s significant potential in modulating disgust, a critical and often debilitating component of mental disorders like SP and OCD. Unlike traditional ET that primarily focuses on fear and avoidance behaviors, iVRET offers a unique avenue for directly addressing complex emotions such as disgust.

Our findings suggest that iVRET can effectively evoke and modulate disgust in controlled environments, enabling patients to confront and habituate to disgust-related stimuli in a safe and controlled manner. This is particularly relevant for conditions where disgust plays a pivotal role in maintaining symptomatology, such as in OCD and SP, where contamination fears are prevalent. As observed in our recent publications [6,53], disgust has evolved from a mechanism primarily aimed at avoiding physical contaminants to one that also encompasses moral and social aspects. These additional dimensions are crucial for understanding the avoidance behaviors observed in SP and OCD. From a neurobiological perspective, we highlight the critical roles of brain regions such as the insula and limbic structures, which are integral in disgust processing. These areas play a key role in integrating multi-sensory data and are associated with the abnormal processing patterns typical of these disorders. Neuroimaging results, as reported by Vicario et al. [53], suggest that disruptions in these neural circuits may contribute to the increased sensitivity to disgust observed in these clinical conditions.

Moreover, the broad spectrum of studies reviewed, encompassing both clinical and healthy populations, underscore iVRET’s versatility and potential for widespread application in disgust management. The reviewed evidence supports the idea that iVRET can produce significant changes in disgust and related avoidance behaviors, marking a crucial step forward in ET’s evolution, and offering a more nuanced and comprehensive treatment approach.

The effectiveness of iVRET in addressing disgust, particularly within the context of contamination fears, has been analyzed in several studies on both healthy [25,26,27,28,29] and clinical populations [17,18,19,20,21,22,23,24]. Among the studies conducted on healthy populations, investigations by Inozu et al. [25,26] stand out, demonstrating VRET’s potential to significantly reduce contamination fear, disgust, and hand-washing urges; in particular, we found large effects size (see Table 2) for iVRET effectiveness on disgust outcomes in the experimental group compared to controls. These studies used VR scenarios that progressively escalated in dirtiness and disgust across different settings, showing that VRET can effectively alleviate both the emotional and behavioral symptoms associated with contamination fears. Importantly, disgust was identified as a mediator in the relationship between contamination fear and washing urges [26], emphasizing its central role in the phenomenology of contamination-related disorders. These findings highlight VRET’s capacity not only to alleviate anxiety and behavioral anomalies but also to directly impact disgust sensitivity, representing a significant advancement in our understanding of how VR can be used to treat complex emotional responses.

Furthermore, Ammann et al. [27] investigated whether VRET could effectively elicit disgust and how this emotion impacts participants’ willingness to eat chocolate in a VR environment. Exposure to a disgust-inducing scenario reduced their inclination to eat chocolate, emphasizing disgust sensitivity’s effect on behavior and the mediating role of physical presence in VR. Also, the study by Dozio et al. [28] highlighted the VR environments’ ability to evoke significant emotional responses, including disgust, although it was not directly focused on therapeutic applications for disgust. The study contributes to the discussion on VR’s effectiveness in eliciting specific emotions, suggesting paths for future research in VRET for emotional responses. Although Minns et al. [29] did not directly measure outcomes on disgust emotion, the authors demonstrated VRET’s significant reduction in spider fear, with substantial improvements in approach behavior and fear measurements. Despite the study’s focus on fear, the high levels of disgust experienced during VR exposure suggest VRET’s broader potential in addressing disgust reactions.

Regarding clinical populations, the reviewed studies demonstrated the efficacy of iVRET in reducing both fear and disgust responses in individuals with SP. Through simulated encounters with virtual spiders, iVRET effectively induced avoidance behaviors and physiological responses indicative of negative emotions, including fear and disgust. Studies such as Binder et al. [18] and Tardif et al. [19] highlighted a significant reduction in avoidance behaviors and physiological activation associated with spider phobia, emphasizing the potential of iVRET as a significant tool in SP treatment. Despite variations in its impact on disgust across different studies, the overall findings suggest that iVRET holds promise as an effective intervention for SP. The emergence of VRET offers a groundbreaking approach for treating OCD, with a particular focus on contamination-related symptoms. Studies such as Belloch et al. [20] and Benzina et al. [21] demonstrate the feasibility and efficacy of immersive VRET in inducing emotional responses for OCD treatment. These investigations underscore patients’ successful adaptation to anxiety-inducing virtual scenarios, resulting in significant reductions in OCD symptoms and enhancements in quality of life. Additionally, research by Cullen et al. [22] compares VRET with conventional exposure methods and indicates comparable effectiveness in provoking anxiety profiles, emphasizing the potential of VRET as a complementary therapy for OCD. Despite the promise shown by VRET in treating anxiety disorders such as SP and OCD with contamination symptoms, several challenges and areas for further research exist.

Studies like Miegel et al. [17] highlight the persistence of obsessions and diverse individual responses in VRET sessions, suggesting the necessity for personalized interventions. Moreover, variability in the impact of VRET on disgust across different studies underscores the importance of exploring individual patient needs and cognitive factors to enhance VRET’s efficacy. Further, limitations such as the lack of follow-up studies to assess long-term effects and small sample sizes hinder the generalizability of findings. Therefore, research is needed to address these challenges and optimize VRET protocols for improved therapeutic outcomes.

The focus of this systematic work is the possibility of targeting disgust emotion with VRET, instead of more generic fear, starting from the idea that this emotion can be considered a transdiagnostic index shared by many psychiatric and neurological disorders, especially for mental diseases with a strong component of fear of contamination [4,5,6]. To the best of our knowledge, we have observed a limited number of studies that specifically examine the evaluation of disgust emotion. However, we believe that disgust is a crucial component of anxiety symptoms and contributes significantly to the experienced distress, particularly in contaminated environments, both for clinical and healthy populations. Therefore, we advocate for more research attention to be directed towards addressing disgust emotion in various contexts to improve our understanding of its role in mental health and well-being. Aligned with our perspective, other studies discussed the limitation of focusing solely on anxiety symptoms, without considering the role of disgust. Mason and Richardson [54] and, more recently, Garcia-Batista [55], underscore the significant contribution of disgust to the development of the manifestation of contamination and washing symptoms in C-OCD. This emotion is often more resistant to treatment than fear, posing challenges to traditional ET. Mason and Richardson [54] note that while ET can be effective for anxiety disorders, its effectiveness in treating disgust-based symptoms is less clear. They suggest that ET might need to be modified or combined with other therapeutic approaches to effectively target disgust. Importantly, although the paper [54] does not specifically discuss VRET, it emphasizes the need for more research into effective treatments for disgust in anxiety disorders, suggesting that future studies could explore innovative methods like iVRET.

The present systematic work aimed to bridge this gap, providing more quantitative evidence on the effectiveness of targeting disgust emotion with iVRET. While iVRET appears promising in reducing anxiety symptoms in general, our analysis revealed a scarcity of studies delving deeply into this theme to facilitate meta-analysis. Therefore, future research with a more focused approach and enhanced statistical rigor on the component of disgust is warranted.

## 5. Conclusions

In this systematic review, we propose a paradigm shift in the treatment of certain mental disorders, particularly those where disgust plays a significant role. The efficacy of iVRET in eliciting and managing anxiety responses in both healthy and clinical populations marks a significant advancement in therapeutic interventions. We highlight the importance of future studies to explore the effectiveness of VR scenarios in evoking disgust emotions. However, it is important to acknowledge the limitations of the present study. The inclusion of only a limited number of papers meeting our criteria, with eight articles exploring the effects of VRET in clinical populations and five in healthy individuals, the small sample sizes of the selected studies (i.e., inclusion of single case), and the exclusion of other clinical categories that could benefit from iVRET, like PTSD or eating disorders, prevents us from drawing robust evidence on this important topic. Nonetheless, the insights gained from this study advocate for the exploration of innovative therapeutic approaches. The potential modification of ET to specifically target disgust, possibly through the integration of iVR, offers a promising avenue for enhancing treatment outcomes for individuals with anxiety disorders characterized by abnormalities in disgust sensitivity. In conclusion, while this study validates the effectiveness of VRET in reducing anxiety symptoms associated with contamination fears, it also highlights new research pathways. Exploring the role of disgust within VRET contexts could revolutionize treatment strategies for OCD and disorders rooted in contamination fear, leading to more comprehensive and effective interventions. Future directions could go deeper into integrating multisensory inputs into iVRET, thereby enhancing its effectiveness by creating more realistic and immersive therapeutic environments. Additionally, the implementation of adaptive algorithms represents another exciting frontier. These algorithms could dynamically adjust the therapy’s intensity and complexity [9] based on real-time feedback from physiological and behavioral responses of the user. Such a personalized approach could optimize exposure levels, ensuring that patients are neither underwhelmed nor overwhelmed. This would help maintain an optimal level of engagement and therapeutic challenge, thereby maximizing the effectiveness of iVRET.

## Figures and Tables

**Table 2:** List of included studies on healthy (top panel) and clinical populations (bottom panel). In columns are variables of interest: Items, authors and years of the selected studies; VR, the type of virtual reality (if immersive, I, or semi-immersive, Semi-I); Type, the typology of the study, if randomized trials or not, or a single case study; Design, the type of experimental design, between (btw), with-in, or mixed designs; VRET Type, design of the VRET intervention used in the study; Disgust Outcome, specification of the disgust outcome measured in the study. The columns Ex. G and C represent the numerosity of samples for experimental and controlled groups, respectively, while the columns Metrics Ex. G and C contain means and standard deviations for the listed disgust outcome. In column *d*, the effect size of the VRET intervention efficacy in experiments with respect to the control group is reported, where possible, while in the last column, the main results and some limitations of the study are reported.

Healthy Population												
Items		VR	Type	Design	VRET Type	Disgust Outcome	Ex. G	Metrics Ex. G	C	Metrics C	*d*	Main Results and Limitations
[27]		I	RCT	btw	VR setting where participants encountered either a neutral scene or one designed to evoke disgust (a regular chocolate piece versus a dog appearing to defecate chocolate). Afterwards, they were requested to eat actual chocolate in the real world.	DS: FDS short	50	Baseline: 3.17 ± 0.89 Post-intervention (who refused to eat the chocolate): 3.60 ± 0.82 Post-intervention (who were willing to eat the chocolate): 3.02 ± 0.87	50	Baseline: 2.96 ± 0.79.	-	The study found that VR can trigger disgust and reduce the desire to eat chocolate. Limitations concern only young participants being tested with non-clinical visuals and touch but without measuring physical responses like facial muscle movements.
[28]		Semi-I	Case study	with-in	VR scenarios designed to evoke various emotions, including disgust.	9-point SAM	48	Baseline: Valence: 6.5 ± 1.13; Arousal: 3.6 ± 1.87; Dominance: 6.21 ± 1.68 Disgust: Valence: 4.15 ± 1.87; Arousal: 4.56 ± 2.11; Dominance: 5.54 ± 2.20	No	-	-	The study outlines a way to create VEs that trigger certain emotions like disgust and evaluates these scenarios. It aims to generate emotions rather than treat anxiety or phobias and does not offer effectiveness data for VRET.
[25]		I	RCT	btw	A structured psychoeducation session about contamination fear and VR-based exposures. There were four scenarios designed to incrementally increase levels of contamination anxiety, disgust, and an urge to wash hands (rated pre- and post-VRET).	DS: DS-R	9	Pre-test: 76.56 ± 10.35, Post-test: 73.33 ± 10.07	12	Pre-test: 72.33 ± 10.33 Post-test: 77.00 ± 10.96	0.79	VRET significantly lessened anxiety, disgust, and hand-washing urges in those with a fear of contamination, showing notably lower scores in these areas versus the control group. However, the study had a small, non-clinical group and didn’t use physiological measures like facial EMG to assess disgust.
[26]		I	RCT	btw	VR scenarios with different levels of dirtiness. The phases were as follows: Training Scenario: familiarization with VR. Scenario 1: kitchen interaction. Scenario 2: cleaning and eating. Scenario 3: handling contaminated items. Scenario 4: trash bin interaction.	Likert’s scale (100 points)	33	HCF Group: Scenario1: 40 ± 20; Scenario2: 55 ± 25; Scenario3: 65 ± 20; Scenario4: 75 ± 20.	33	LCF Group: Scenario1: 20 ± 10; Scenario2: 30 ± 10; Scenario3: 40 ± 10; Scenario4: 50 ± 10.	6.42	The study found that VR effectively provoked anxiety, disgust, and washing urges across different fear levels, intensifying with the VR’s dirtiness. This suggests VR’s potential in exposure therapy, particularly for contamination fear in OCD. Limitations include the non-clinical sample and no physiological measures.
[29]		I	RCT	btw	A single session of iVRET, which included six 5 min exposures to stereoscopic 3D videos of spiders, delivered through a VR headset.	BAT	38	Pre-treatment: 79.40 ± 18.77 Post-treatment: 47.03 ± 26.89	39	Pre-treatment: 70.44 ± 17.63 Post-treatment: 60.00 ± 24.281	0.47	iVRET proved to significantly lower anxiety and spider phobias, outperforming psychoeducation by reducing FSQ and BAT scores. More research is needed on disgust and clinical validation.
**Clinical Population**												
	**Items**	**VR**	**Type**	**Design**	**VRET Type**	**Disgust Outcome**	**Ex. G**	**Metrics Ex. G**	**C**	**Metrics C**	***d***	**Main Results and Limitations**
**SP**	[18]	I	RCT	mixed	The three VR scenarios were as follows: 1. Fishing, a behavioral search task; 2. Path-Choice, a forced-choice task; 3. Touch the Enemy, a behavioral approach task.	DS:FEE; FEE correlations with FSQ	15	Baseline Phobic: 3.37 ± 0.58	10 6	Baseline non-fearful: 2.88 ± 0.53 Baseline fearful: 3.44 ± 0.76	-	FEE scores correlate with FSQ scores, especially in the body secretion and hygiene category, but show an inverse relationship with oral rejection and spider dislike. This indicates iVRET may reduce disgust as spider fear decreases. Study limitations include a small, all-female sample, suggesting a need for broader future research to validate findings and assess VRET’s long-term effects.
	[24]	I	RCT	btw	VR consisted of navigating a virtual house and interacting with stimuli representing OCD subtypes: contamination/cleaning, checking, symmetry, and hoarding.	DS: FEE; FEE correlations with SUDS (0.22)	29	Baseline: 7.25 ± 25.15	29	Baseline: 79.22 ± 14.80	-	Quetiapine showed a notable decrease in somatic anxiety, indicating it acts quickly to ease anxiety. Correlations suggest that there is some degree of association between baseline disgust sensitivity (as measured by FEE) and the participants’ responses in various aspects during the challenge (e.g., anxiety levels and discomfort). It is in doubt if an artificial environment would have anxiolytic effects per se or might just increase arousal and fear.
	[19]	I	RCT	with-in	VRET consisted of three levels of sensory simulations: visual, tactile, and haptic feedback.	TD: ESF	59	Pre-immersion: VIS = 93.21 ± 14.27; VIS + TACT = 96.35 ± 11.48; VIS + TACT + HAPT = 86.45 ± 21.07. Post-immersion: VIS = 73.74 ± 15.37; VIS + TACT = 75.95 ± 15.05; VIS + TACT + HAPT = 73.05 ± 24.38.	No	-	1.70	Although disgust changes were tracked, they did not strongly predict fear reduction, emphasizing the intricate dynamics of phobia and the significance of cognitive factors over physiological or emotional factors. Nevertheless, iVRET showed a substantial effect in modulating disgust. The study’s limitations are a lack of a control group and the performance of only one session.
**OCD**	[20]	I	non-RCT	with-in	VR consisted of Contaminated Virtual Environment (COVE) tasks to induce anxiety and disgust. The VR environments consisted of different actions performed in a dirty kitchen.	Likert’s scale (10 points)	4	Action1: 2.00 ± 0.41 Action2: 3.38 ± 0.75 Action3: 4.38 ± 1.11 Action4: 6.75 ± 0.96	No	-	-	The study assessed the use of VRET for treating OCD, aiming to provoke anxiety and disgust. Participants showed heightened disgust as they moved through actions in VR, indicating VR’s potential in evoking emotions relevant to OCD therapy. However, with only four female participants and no control group or varied conditions, the findings’ validity and applicability are limited.
	[21]	I	non-RCT	single case	Personalized iVRET involved 15 weekly sessions of exposure to 360° immersive videos filmed from the patient’s perspective in her own environment, using a VR headset.	Disgust reaction as peak in skin conductance (SC).	1	missing	No	-	-	The study showed that individualized iVRET significantly helped a patient with severe OCD, reducing symptoms and enhancing quality of life. This was seen in clinical assessments and skin conductance measures. While not directly measured, reduced avoidance behavior suggests less disgust. Due to being a single case study, further research with more subjects is necessary to generalize the results.
	[22]	I	RCT	with-in	The two VR sessions were as follows: 1. An instruction phase that prepares participants for the exposure task, triggering anticipatory stress. 2. A contact phase that has them face anxiety-inducing elements in VR, mimicking the stress of real-life exposure to contaminants.	Disgust differences between “Instruction” and “Contact” phases based on SUD and physiological measures.	22	missing	No	-	-	The study found personalized iVRET beneficial for a severe, treatment-resistant OCD patient, reducing symptoms and enhancing quality of life. Disgust response improvements were implied but not directly measured. As a single case study, broader conclusions cannot be drawn; larger, controlled studies are needed to confirm these findings and investigate the long-term effects and treatment of disgust with VRET.
	[23]	I	Non-RCT	btw	VRET involved navigating a virtual house and interacting with a standardized set of 10 stimuli representing OCD subtypes: contamination/cleaning, checking, symmetry, and hoarding.	SUD	44	Post-intervention: 14.74 ± 11.09	31	Post-intervention: 4.39 ± 5.73	4.76	The study found significant differences in distress and behaviors between OCD patients and healthy controls, highlighting the potential of iVRET, as showed by the large effect size. Some limitations concern a fixed stimulus sequence and some incomplete data.
	[17]	I	RCT	with-in	Multiple VRET sessions were performed over six weeks, with the assessment of subjective distress and physiological responses to measure arousal and disgust.	SUD	8	SUD baseline: 33.12 ± 26.31 SUD peak: 67.50 ± 24.93	No	-	1.11	The study found that Virtual Emotion Regulation Psychotherapy (VERP) triggered distress and arousal in C-OCD patients, with disgust being the central emotion addressed. Some reduction in OCD symptoms was noted, showing medium to large effect sizes, but only two out of eight patients significantly responded to the treatment. The study’s reliability is affected by its small sample size and lack of a control group, which may affect the applicability of the results to a broader population.

Legend. DS: Disgust Sensitivity; TD: Trait Disgust; DS-R: The Disgust Scale-Revised; FDS short: Food Disgust Scale; SAM: Self-Assessment Manikin; SUD: Subjective Units of Distress; FEE: Disgust Sensitivity Scale; ESF: Trait Disgust Scale; FSQ: Fear of Spiders Questionnaire; EMG: Electromyography; BAT: behavioral approach test; d: Cohen’s *d*; the columns Ex. G and C represent numerosity of experimental and controls groups, respectively. Reported values in Metrics are in the form of mean values ± standard deviations.
